# Supplementary figures and images for: Plant phenomics & precision agriculture simulation of winter wheat growth by the assimilation of unmanned aerial vehicle imagery into the WOFOST model
Source: PLoS One. 2021 Oct 8;16(10):e0246874. doi: 10.1371/journal.pone.0246874 (PMC8500443; doi:10.1371/journal.pone.0246874)

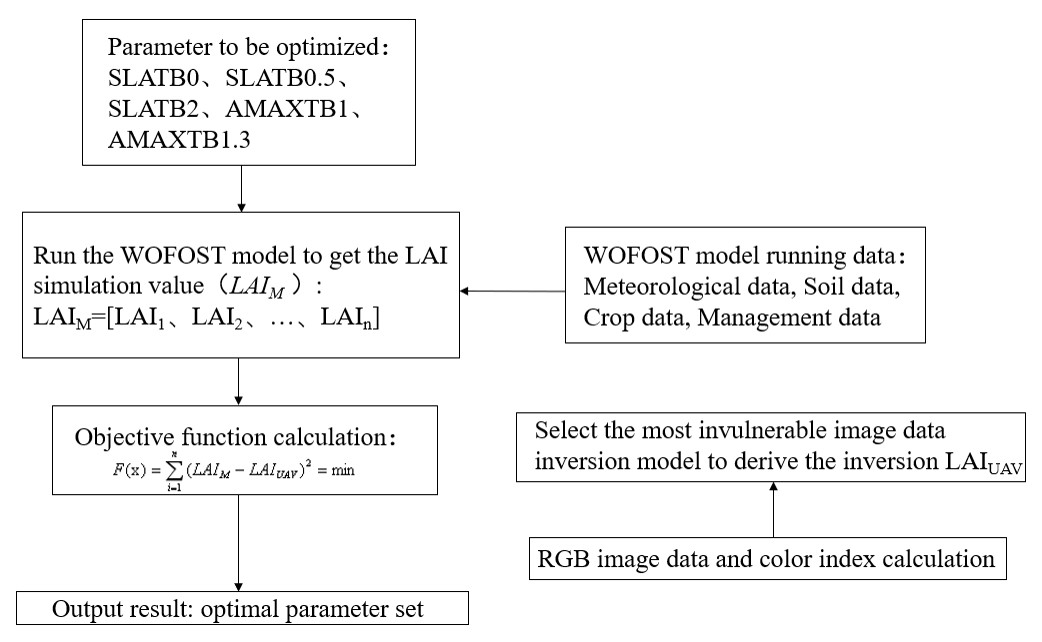

Supplement: S1 Fig — (TIF) [file pone.0246874.s001.tif]

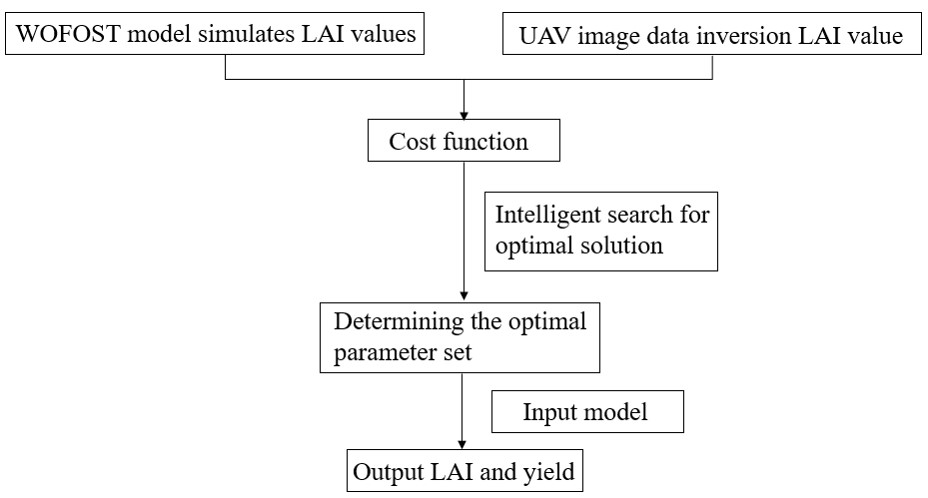

Supplement: S2 Fig — (TIF) [file pone.0246874.s002.tif]

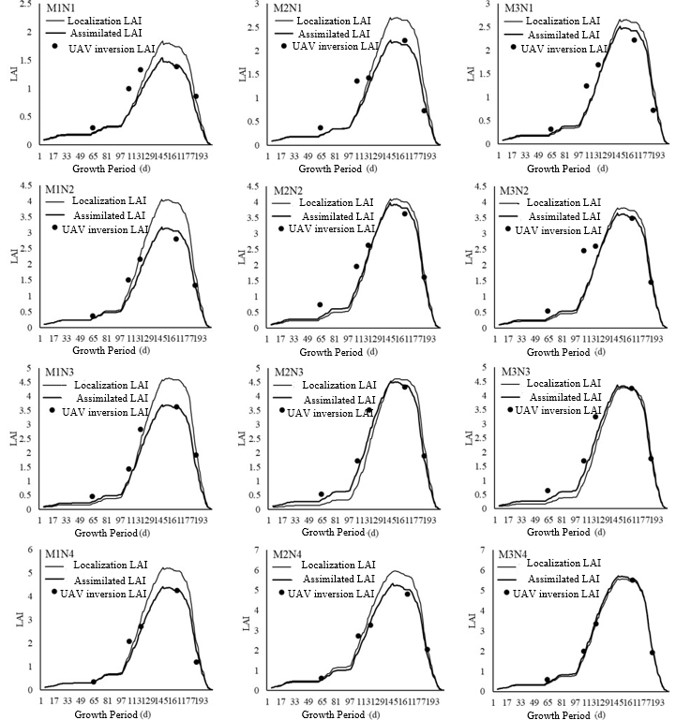

Supplement: S3 Fig — (TIF) [file pone.0246874.s003.tif]

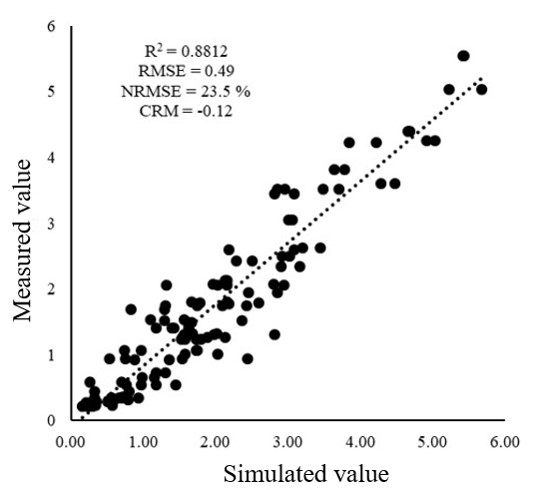

Supplement: S4 Fig — (TIF) [file pone.0246874.s004.tif]

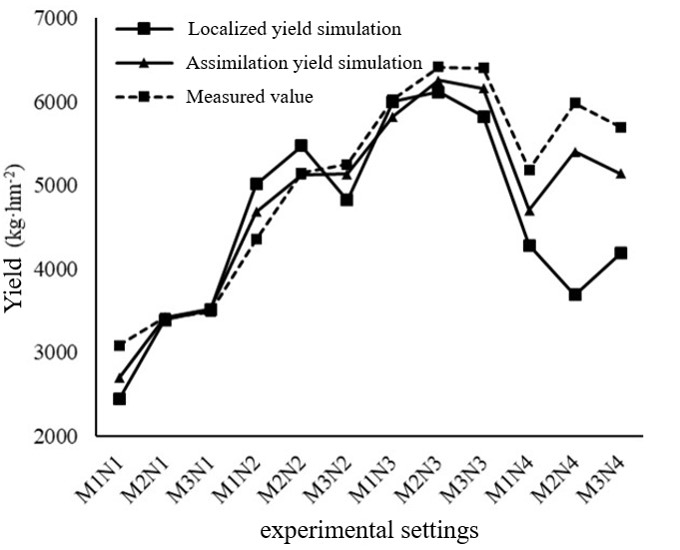

Supplement: S5 Fig — (TIF) [file pone.0246874.s005.tif]

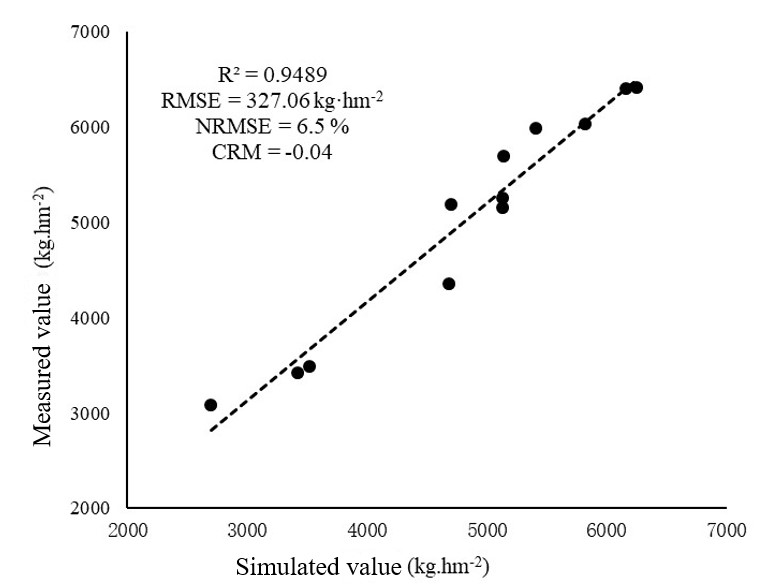

Supplement: S6 Fig — (TIF) [file pone.0246874.s006.tif]
