# Supplementary material for: Plant phenomics & precision agriculture simulation of winter wheat growth by the assimilation of unmanned aerial vehicle imagery into the WOFOST model
Source: PLoS One. 2021 Oct 8;16(10):e0246874. doi: 10.1371/journal.pone.0246874 (PMC8500443; doi:10.1371/journal.pone.0246874)
Supplement: Attachment — Submitted filename: Response to Reviewers.docx [file pone.0246874.s008.docx]

**Response to Comments**

Reviewer #1: his paper represents a study of simulation of winter wheat growth by the assimilation of unmanned aerial vehicle imagery into the WOFOST model at the finer spatial resolution. And readers are very interested in the topic. Generally, this paper is well written, some comments can be found as followed,

(1)Suggest conduct an sensitivity analysis to determine the optimized parameters, I think that specific leaf area (SLATB0, SLATB0.5, SLATB2) and maximum CO2 assimilation rate (AMAXTB1, AMAXTB1.3) is not suitable for reinitialized because CO2 assimilation rates are relative constant values for wheat.

**R:** The parameters for initialization adjustment in this article are all determined by the results of sensitivity analysis. Taking into account the local varieties and cultivation measures, compare the leaf area and the maximum CO2 assimilation rate (AMAXTB1, AMAXTB1.3) for reinitialization to make the results more accurate.

(2) I didn’t see how the retrieve the LAI from UAV imagery and evaluation of accuracy of LAI. More texts need to be added to clarify this point.

**R:** The extraction of LAI from UAV images and the content of LAI accuracy evaluation have been explained in detail in another article.

(3) References

**R:** Replaced

Reviewer #2: Review for Plos One

1. Introduction

It is written in lines 48-50 that the parameter optimization method is superior in terms of assimilation results. This statement should be clearly explained and supported by a bundle of citations. To the knowledge of the Reviewer, other methods such as updating method using EnKF have quite high performance especially in the consideration of the measurement uncertainties. In addition, the operability of the proposed assimilation method should be adequately discussed? When do we acquire data to assimilate them during the growing season? The originality of this contribution relies on the fact that, although UAV is widely used in crop monitoring, most studies related to assimilation methods use high-altitude remote sensing (RS) and not UAV data. The interest of UAV-based RS should be better highlighted in terms of scales, operability, spatial and temporal resolution with respect to, for instance, satellite-based RS. In the introductive section, the proposed research work should be better detailed and supported by the state of the art in order to specify the scientific objectives with regard to the considered material and methods. For the Reviewer, the formulation of the paper objectives is too general.

**R:** (1) The parameter optimization method mentioned in this article is actually a general term for EnKF, SAA, and ordinary least squares. In the research, ordinary least squares were selected. (2) This study mainly carried out data acquisition in the five key stages of wheat growth: jointing stage, heading stage, flowering stage, filling stage, and mature stage.

2. Material and methods

The material and methods section appears not complete enough to better understand the research scope and limitations of the proposed method. Why two experiments are considered in response to the research objectives? The measurement methods for LAI and crop yield are not described at all, which renders difficult the interpretation of the results. Also, please give the crop density in terms of grains per meter square. Although WOFOST is quite known in the community, the key principle of modelling, the considered assumptions and values of major parameters (line 191) should be given for the sake of completeness and research reproducibility. Also, please give the definition of R^2^ in Equation 1. The extraction of LAI from UAV imaging is not described. This is a critical point to explain, present and discuss in terms of methodology, operability and accuracy. What are the sensors used? What is the altitude? What is the regression? … It is not clear for the Reviewer when data assimilation is performed. Is it at each remotely sensed LAI? If so, this should be better highlighted via a Figure. Is it necessary to perform assimilation at all measurement point? Equation 4 and 5 are not mathematically valid. Please adapt them. Line 199, please correct “A comparison in made in …” Line 203, please define earlier in the paper the notion of localized model. Line 227, please give the definition of the confidence interval. Line 273, CRM is negative

**R:** (1) The data of the second year is used to verify the results; (2) The leaf area index measurement method: 15 plants were sampled in the experimental plot, and the length and width coefficient method was used to determine the leaf area, and then the specific leaf weight method was used to calculate the leaf area index. Yield measurement: In the mature period of wheat, the number of ears per unit area was investigated in each plot, and 30 single ears were taken for indoor experiment to calculate the three factors of yield (number of ears, number of grains per ear, and thousand-grain weight). Take 1m2 area of wheat from each plot, thresh and test the yield. (3) In this study, the inspire 1RAW drone produced by DJI is equipped with a Zenmuse X5R aerial camera for data collection. The flying height of the drone is 9 meters. (4) What is regression (5) The localized model refers to the adjustment of related parameters so that the model can simulate the growth of wheat in the study area. (6) This study mainly carried out data acquisition and assimilation in five key stages of wheat growth: jointing stage, heading stage, flowering stage, filling stage, and mature stage.

3. Discussion

The scale of the study should be criticized. The Authors present their model as valid at the regional scale. Is it realistic with UAV technology?

**R:** The combination of UAV remote sensing and WOFOST model is applied to the simulation of crop agronomic parameters. This method is still in the exploratory stage. Cheng et al. used a combination of multispectral UAV and WOFOST model to estimate corn yield. This proves that this method is feasible. (Cheng Z, Meng J, Shang J, et al. Generating Time-Series LAI Estimates of Maize Using Combined Methods Based on Multispectral UAV Observations and WOFOST Model[J]. Sensors, 2020, 20(6006):6006.)
